# Relationship of early acute complications and insertion site in push method percutaneous endoscopic gastrostomy

**DOI:** 10.1038/s41598-020-77553-6

**Published:** 2020-11-25

**Authors:** Hiroshi Suzuki, Satoru Joshita, Tadanobu Nagaya, Koichi Sato, Akihiro Ito, Tomoaki Suga, Takeji Umemura

**Affiliations:** 1 Department of Gastroenterology, Division of Medicine, Shinshu University School of Medicine, Asahi 3-1-1, Matsumoto, Nagano 390-8621 Japan; 2 Department of Gastroenterology, Matsumoto City Hospital, Matsumoto, Japan; 3 Department of Life Innovation, Institute for Biomedical Sciences, Shinshu University, Matsumoto, Japan

**Keywords:** Gastroenterology, Risk factors, Nutrition disorders

## Abstract

Percutaneous endoscopic gastrostomy (PEG), which is frequently used for nutrition management in patients having difficulty with oral intake, is considered a safe procedure. However, serious complications may occur depending on site of the puncture. This study aimed to clarify whether push method PEG construction at the posterior wall (PW) of the greater curvature (GC) had a higher risk of complications. We retrospectively investigated the relationship between puncture site at the PW of the GC and early acute complications in 540 patients receiving PEG. Early acute complications were defined as bleeding or perforation within 30 days after the PEG procedure. PEG-related complications were observed in 80 patients in total, with early acute complications detected in 42 patients. PEG construction at the PW of the GC in 12 cases exhibited a significantly higher occurrence of early acute complications versus PEG at other sites (41.7% vs. 7.0%, *p* = 0.001). Further, multivariate analysis revealed PW at the GC to be independently associated with early acute complications (OR 9.59, 95% CI 2.82–32.61; *p* = 0.0003). It may be desirable to avoid PEG at the PW of the GC. If performed, clinicians should pay careful attention to early acute complications.

## Introduction

First performed by Gauderer et al. in 1980^1^, percutaneous endoscopic gastrostomy (PEG) is a medical procedure to provide enteral nutrition for patients having difficulty with oral nutrient intake^2^. PEG has no inferiority to surgical gastrostomy in terms of morbidity or mortality^3^, with success rates of 95–100%^4^. Anderloni et al. assessed early and late (30-day) complications and mortality for PEG. The 30-day mortality rate was 1.8% and complications were detected in 1.7% of patients, which supported the safety of the PEG procedure^5^. However, the most frequent, albeit non-serious, complication was infection (50%), followed next by bleeding (32.1%), tube dislodgement (14.3%), and buried bumper syndrome (3.6%)^5^. Thus, serious complications may occur depending on the type and location of the puncture^6,7^. To the best of our knowledge, few reports have addressed the relationship between PEG site and complications. Lee et al. found that PEG tube insertion in the upper body of the stomach was a significant risk factor for early and late complications by multivariate analysis^8^. Those complications were suspectedly caused by relatively long distances between the gastric and abdominal walls for the upper body as compared with those for the lower body, which might have produced stronger tension between the abdominal and gastric walls during stomach contraction to induce slow or incomplete fistula formation^8^.

**Figure 1:**
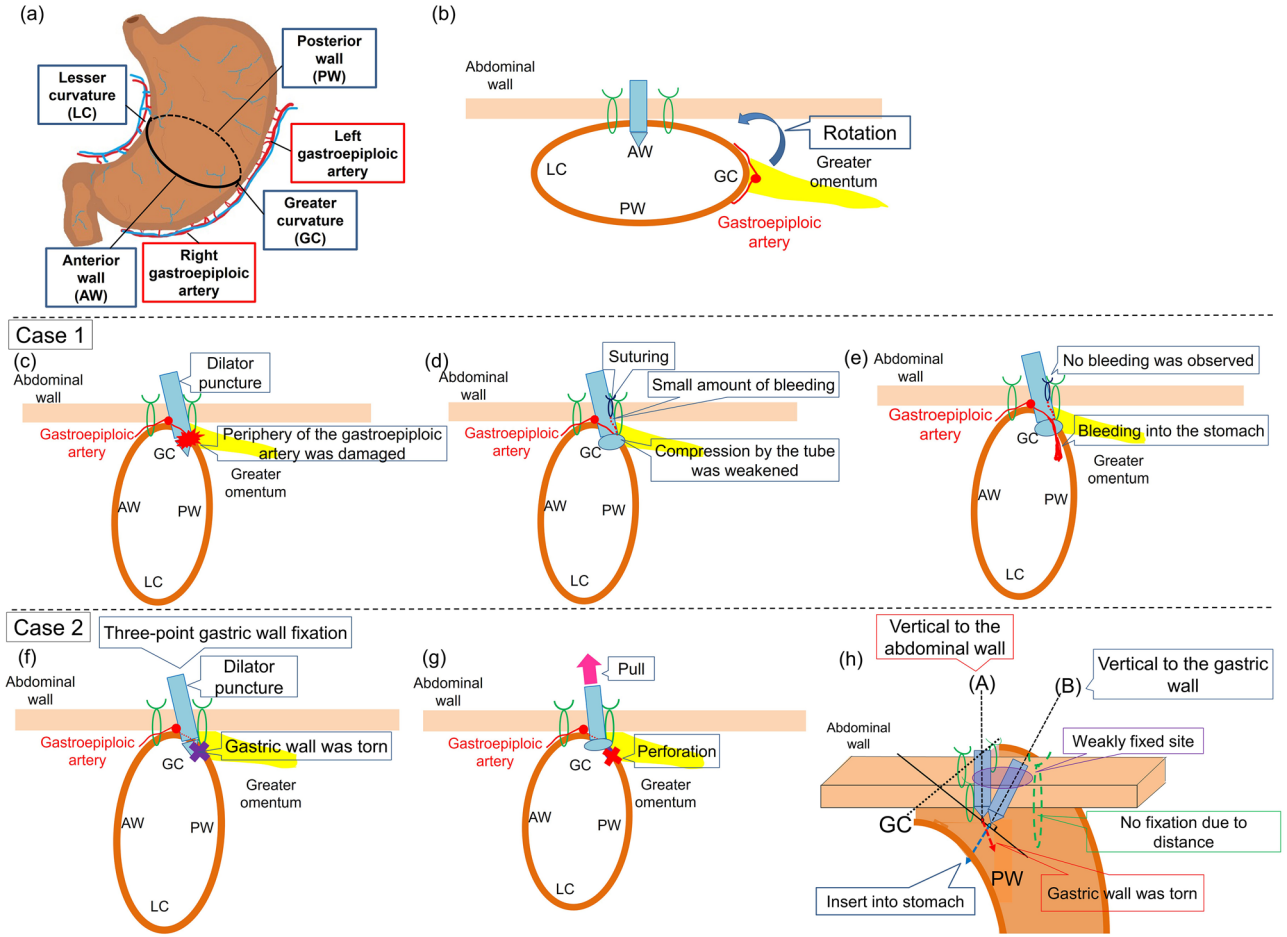
Anatomy of the arteries around the stomach and schematic diagram explaining the cause of complications in case 1 and case 2. (**a**) Anatomically, the left and right gastroepiploic arteries are located through the greater omentum on the GC side. (**b**) Short-axis image of the stomach. The PEG tube usually safely punctures the AW of the stomach. However, if the stomach is rotated in the long axis, it is difficult to insert the tangential dilator towards the AW of the middle body of the stomach. (**c**) It was believed that the periphery of the gastroepiploic artery was damaged by a puncture in the PW of the GC. (**d**) A small amount of bleeding was intermittently observed in the patient’s gauze dressing. Compression by the tube was weakened due to subcutaneous suturing at the insertion site. (**e**) Bleeding into the stomach occurred through the fistula. (**f**) When the stomach was rotated in the long axis, the dilator puncture direction became tangential to the PW of the stomach. At that time, we suspected damage of the stomach wall. (**g**) The damaged stomach wall became torn due to traction compression for bleeding after PEG insertion. (**h**) Since the PW of the GC was curved, the PW was too long to fix the stomach wall. When the dilator was inserted vertically into the abdominal wall as shown in (**h**)-(A), the puncture force was not transmitted vertically to the stomach wall, and the dilator entered the stomach while tearing the stomach wall to the PW side. On the other hand, in order to insert the dilator vertically into the stomach wall as in (**h**)-(B), it is necessary to puncture the abdominal wall obliquely. In case 2, the inserted tube was seen endoscopically as towards the PW and was considered to have followed the path shown in (**h**)-(A).

Gastrostomy should generally be made at the gastric anterior wall (AW) (Fig. 1a,b). However, when the AW of the stomach is far from the abdominal wall due to stomach rotation, the greater curvature (GC) can be selected for introducer modification of PEG tube insertion (Fig. 1a,b). After encountering two recent cases of serious complications following PEG by introducer modification at the posterior wall (PW) of the GC, we hypothesized a higher complication rate for PEG placement at the PW of the GC. To examine this notion, we retrospectively investigated the relationship between puncture site and complications in patients receiving PEG and described the clinical outcomes of the two cases.

## Results

### Patient background

The clinical characteristics of all patients are summarized in Table 1. A total of 374 patients (69.3%) were male and median age was 72 years. Anti-platelet or anti-coagulant agents were given to 118 patients. PEG with push method insertion was indicated for such background diseases as head and neck cancer, neurological disease, cerebrovascular disease, and dementia in all cases. Forty-two early acute complications, including four severe acute complications, were noted.

**Table 1:** Patient background and univariate analysis of PEG site (n = 540).

Characteristic, median (range)	All cases (n = 540)	PW of GC group (n = 12)	Other site group (n = 528)	Univariate		Multivariate**	
				*p*-value	OR (95% CI)	*p*-value	OR (95% CI)
Age (years)	72 (17–99)	68 (44–91)	72 (17–99)	0.674	–		
Male, n (%)	374 (69.3)	11 (91.7)	363 (68.8)	0.089	5.0 (0.64–39.0)		
BMI	18.4 (11–35.4)	19.9 (12.8–24)	18.4 (11–35.4)	0.646	–		
Anti-platelet or anti-coagulant agents, n (%)	118 (21.9)	4 (33.3)	114 (21.6)	0.330	1.82 (0.54–6.14)		
**Background disease**							
Head and neck cancer, n (%)	215 (39.8)	5 (41.7)	210 (39.8)	0.895	1.08 (0.34–3.45)		
Other including neurological, cerebrovascular, and dementia, n (%)	325 (60.2)	7 (58.3)	318 (60.2)				
Esophageal hernia, n (%)	96 (17.8)	5 (41.7)	91 (17.3)	0.029	3.41 (1.06–11.0)	0.042	3.39 (1.04–11.03)
**Laboratory data**							
WBC (/µL)	6230 (2410–19,840)	6945 (3320–8630)	6210 (2410–19,840)	0.843	–		
Hemoglobin (g/dL)	12.1 (6.6–17)	12 (8.7–15.3)	12.1 (6.6–17.0)	0.950	–		
Plt (10^4^/μL)	23.9 (3.3–64.9)	26.8 (19–36.3)	23.7 (3.3–64.9)	0.155	–		
Albumin (g/dL)	3.4 (1.7–4.9)	3.3 (2.5–4.2)	3.4 (1.7–4.9)	0.857	–		
CRP (mg/dL)	0.52 (0–19.9)	0.85 (0.02–3.57)	0.5 0–19.9)	0.973	–		
PT% (%)	88.5 (7.1–144.3)	85.7 (46.7–105)	88.5 (7.1–144.3)	0.394	–		
APTT (s)	29 (19.4–180)	29.7 (23.9–43.9)	29 (19.4–180)	0.749	–		
Operator experience (years)	7 (3–33)	6 (3–9)	7 (3–33)	0.030	–	0.058	0.73 (0.52–1.01)
Number of gastropexies*	39 (7.2), 168 (31.1), 327 (60.6), 6 (1.1)	3 (25), 2 (16.7), 7 (58.3), 0 (0)	36 (6.8), 166 (31.4), 320 (60.6), 6 (1.2)				
Three-point gastropexy	168 (31.1)	2 (16.7)	166 (31.4)	0.274	0.44 (0.10–2.01)		
Four-point gastropexy	327 (60.6)	7 (58.3)	320 (60.6)	0.873	0.91 (0.29–2.91)		
Early acute complications, n (%)	42 (7.8)	5 (41.7)	37 (7.0)	0.001	9.48 (2.87–31.3)		
Severe acute complications, n (%)	4 (0.7)	2 (16.7)	2 (0.4)	0.003	52.6 (6.72–411.6)		

Early acute complications were defined as bleeding and perforation. Severe acute complications were defined as those requiring surgical intervention. The significance of an association was evaluated using the chi-square test. Fisher’s exact probability test was used for groups with fewer than five samples. The Mann–Whitney U-test was employed to analyze continuous variables.

*APTT* activated partial thromboplastin time, *BMI* body mass index, *CRP* C-reactive protein, *GC* greater curvature, *OR* odds ratio, *PEG* percutaneous endoscopic gastrostomy, *Plt* platelet count, *PT%* prothrombin%, *PW* posterior wall, *WBC* white blood cell count.

*Two-point fixation, three-point fixation, four-point fixation, and unknown.

**The two significant univariate analysis factors of esophageal hernia and operator experience were included in multivariate analysis.

### Complications occurring within and later than 30 days after PEG

PEG-related complications were observed in 80 patients. Complications within 30 days after PEG were recorded in 62 patients, among which wound bleeding was the most frequent (32 cases), followed next by wound infection (five cases). Based on the evaluation criteria^5^, there were 42 cases with early acute complications. Complications at 30 days or more after PEG were observed in 18 patients (Table 2). Most wound bleeding cases were successfully treated by compression hemostasis or subcutaneous suturing. Of the four cases of severe acute complications, two were related to the PEG site at the PW of the GC: one with gastroepiploic artery bleeding (case 1) and the other with gastric perforation (case 2), as described below. The severe acute complications in the remaining two cases were related to the PEG procedure not with the PW of the GC. One patient who was complicated with an abscess as a severe complication case at 30 days or more after PEG received surgical abscess drainage (Table 2).

**Table 2:** Classification of complications (n = 80).

Complications within 30 days after PEG				Complications 30 days or more after PEG	
Infection-related	n	Infection-unrelated	n		n
Wound infection	5	Wound bleeding*	32	Defective granulation	7
Aspiration pneumonia	5	Arterial bleeding*	5	Wound infection	4
Focal peritonitis*	2	Mallory-Weiss syndrome	3	Tube obstruction	3
Subcutaneous emphysema*	1	Tube blockage	3	Skin inflammation	1
		Gastric perforation*	2	Buried bumper syndrome	1
		Buried bumper syndrome	2	Gastric ulcer	1
		Self-extraction	1	Portal emphysema and gastric emphysema	1
		PEG-unrelated death	1		

*PEG* percutaneous endoscopic gastrostomy.

*Defined as an early acute complication based on a reported definition^5^.

### Comparisons of clinical indices in relation to PEG site

In order to clarify the clinical features of cases receiving PEG at the PW of the GC, patients were divided into two groups based on PEG site: the PW of the GC group (12 cases) and the other site group (528 cases). No remarkable differences were observed for complication risk factors between the PW of the GC group and the other site group, including the use of anti-platelet drugs and blood examination data (Table 1), apart from the frequency of esophageal hernia (41.7% vs. 17.3%, OR 3.41, 95% CI 1.06–11.0; *p* = 0.029) and operator experience (6 vs. 7 years, *p* = 0.030). The presence of an esophageal hernia was an independent factor associated with PEG tube insertion at the PW of the GC in multivariate logistic regression analysis (OR 3.39, 95% CI 1.04–11.03; *p* = 0.042) including the two univariately significant factors above. Early acute complications (41.7% vs. 7.0%, OR 9.48, 95% CI 2.87–31.3; *p* = 0.001) and severe acute complications (16.7% vs. 0.4%, OR 52.6, 95% CI 6.72–411.6; *p* = 0.003) were significantly more frequent in the PW of the GC group than in the other site group (Table 1).

### Comparisons of groups with and without early acute complications

In comparisons of clinical indices between early (less than 30 days after PEG) and non-early (30 days or more after PEG) acute complication groups to identify the risk factors for early acute complications of the PEG procedure, the frequency of patients who received anti-platelet drugs was significantly higher in the early acute complication group (35.7% vs. 20.7%, OR 2.13, 95% CI 1.09–4.15; *p* = 0.024), while hemoglobin was significantly lower (11.2 g/dL vs. 12.2 g/dL, *p* = 0.042). The frequency of the PEG site at the PW of the GC was significantly higher in the early acute complication group (11.9% vs. 1.4%, OR 9.48, 95% CI 2.87–31.3; *p* = 0.001) (Table 3). Among the three significant univariate factors above, PEG tube site at the PW of the GC was independently associated with early acute complications (OR 9.59, 95% CI 2.82–32.61; *p* = 0.0003) by multivariate analysis model 1, which included all three parameters (Table 3). PEG tube site at the PW of the GC was also confirmed as an independent factor of early acute complications in models 2 and 3, each containing two of the three factors (Supplementary Table S1).

**Table 3:** Patient background and univariate analysis of early and non-early acute complication groups.

	Early acute complication group (n = 42)	Non-early acute complication group (n = 498)	Univariate		Multivariate**	
			*p*-value	OR (95% CI)	*p*-value	OR (95% CI)
Age (years)	74 (32–89)	71 (17–99)	0.211	–		
Male, n (%)	30 (71.4)	344 (69.1)	0.751	1.12 (0.56–2.25)		
BMI	18.2 (12.8–25.0)	18.4 (11–35.4)	0.885	–		
Anti-platelet or anti-coagulant agents, n (%)	15 (35.7)	103 (20.7)	0.024	2.13 (1.09–4.15)	0.053	1.97 (0.99–3.90)
**Background disease**						
Head and neck cancer, n (%)	13 (31.0)	202 (40.6)	0.221	1.52 (0.77–3.0)		
Other including neurological, cerebrovascular, and dementia, n (%)	29 (69.0)	296 (59.4)				
Esophageal hernia, n (%)	10 (23.8)	86 (17.3)	0.292	1.49 (0.71–3.15)		
**Laboratory data**						
WBC (/µL)	6660 (3600–14,280)	6160 (2410–19,840)	0.318	–		
Hemoglobin (g/dL)	11.2 (6.6–15.3)	12.2 (7–17.0)	0.042	–	0.053	0.86 (0.73–1.00)
Plt (10^4^/μL)	23.6 (3.3–36.0)	23.9 (3.8–64.9)	0.568	–		
Albumin (g/dL)	3.2 (2–4.6)	3.4 (1.7–4.9)	0.164	–		
CRP (mg/dL)	0.62 (0–11.7)	0.51 (0–19.9)	0.622	–		
PT% (%)	89 (14.6–117.2)	88.5 (7.1–144.3)	0.643	–		
APTT (s)	29.9 (20.4–87.4)	28.9 (19.4–180)	0.062	–		
Operator experience (years)	7.0 (3–26)	7.0 (3–33)	0.811	–		
Number of gastropexies*	2 (4.8),11 (26.2), 27 (64.3), 2 (4.8)	37 (7.4), 157 (31.5), 300 (60.2), 4 (0.9)				
Three-point gastropexy	11 (26.2)	157 (31.5)	0.473	0.77 (0.38–1.57)		
Four-point gastropexy	27 (64.3)	300 (60.2)	0.607	1.19 (0.62–2.29)		
PW of GC, n (%)	5 (11.9)	7 (1.4)	0.001	9.48 (2.87–31.3)	0.0003	9.59 (2.82–32.61)
GC side vs. AW side	17 (40.5)	134 (26.9)	0.060	1.85 (0.97–3.53)		

Early acute complications were defined as bleeding and perforation. The significance of an association was evaluated using the chi-square test. The Mann–Whitney U-test was employed to analyze continuous variables.

*APTT* activated partial thromboplastin time, *AW* anterior wall, *BMI* body mass index, *CRP* C-reactive protein, *GC* greater curvature, *OR* odds ratio, *Plt* platelet count, *PT%* prothrombin%, *PW* posterior wall, *WBC* white blood cell count.

*Two-point fixation, three-point fixation, four-point fixation, and unknown.

**In model 1, the three significant univariate analysis factors of anti-platelet or anti-coagulant agents, hemoglobin, and PW of the GC were included for multivariate analysis.

### Relationship between gastropexy number and acute complications

Although it would appear that the risk of blood vessel damage increases with the number of punctures for PEG insertion, this notion has not been addressed to the best of our knowledge. We observed no remarkable differences for three-point and four-point gastropexy in groups with or without early acute complications (three-point gastropexy: 26.2% vs. 31.5%; OR 0.77, 95% CI 0.38–1.57, *p* = 0.473, and four-point gastropexy: 64.9% vs. 60.2%; OR 1.19, 95% CI 0.62–2.29, *p* = 0.607) (Table 3).

### Two cases of early acute severe complications

**Figure 2:**
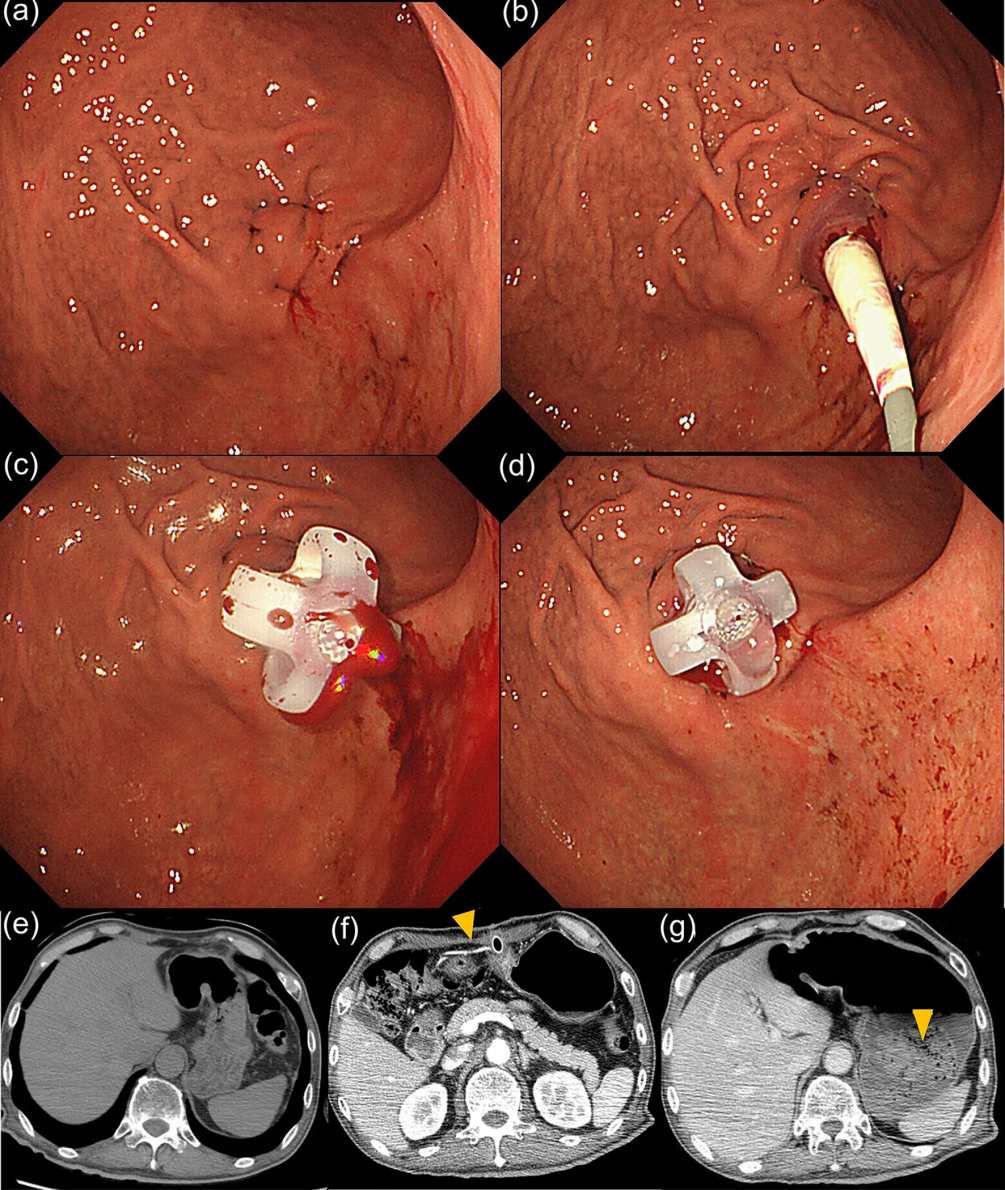
Endoscopic and CT images before and after PEG construction in case 1. (**a**) Four-point fixation was performed. (**b**) The dilator tube was inserted into the PW of the GC. (**c**) Hemorrhage was observed at the time of gastrostomy tube insertion. (**d**) Compression hemostasis was performed by pulling on the tube for 10 min. (**e**) Before PEG construction, CT detected no interfering organs between the stomach and abdominal wall. (**f**) On postoperative day 11, an artery was found near the gastrostomy tube, but no intra-abdominal hemorrhage was observed (arrowheads). (**g**) On postoperative day 11, a hematoma was detected in the stomach (arrowheads).

Case 1 was a 60-year-old male patient who was indicated for PEG due to dysphagia from amyotrophic lateral sclerosis. Blood tests were unremarkable, and computed tomography (CT) detected no interfering organs between the stomach and abdominal wall (Fig. 2e). A finger sign was routinely confirmed. Four-point gastric wall fixation was performed (Fig. 2a), followed by dilator and PEG tube insertion at the PW of the GC (Figs. 1c, 2b). Hemorrhage was observed during insertion (Fig. 2c), for which compression homeostasis was performed by pulling on the tube for 10 min (Fig. 2d). Ensuing blood tests disclosed no anemia, with no bleeding until postoperative day 2. A small amount of intermittent hemorrhage was observed in the patient’s gauze dressing from postoperative day 3 (Fig. 1d). Hemostasis was achieved by pulling on the gastrostomy tube. Contrast-enhanced CT on postoperative day 8 did not indicate a pseudoaneurysm or hematoma. On postoperative day 11, two sutures were made in the skin at the margin of the PEG tube due to continuous bleeding (Fig. 1d). Three hours later, the patient went into shock and required urgent blood transfusion. Contrast-enhanced CT detected no intra-abdominal hemorrhage (Fig. 2f), although a hematoma in the stomach was evident (Figs. 1e, 2g). Emergency surgery revealed hemorrhage from the periphery of the gastroepiploic artery near the fistula, which was promptly ligated. In this case, the periphery of the gastroepiploic artery was presumably damaged during dilator insertion at the PW of the GC (Fig. 1c), likely since the left and right gastroepiploic arteries were anatomically located through the greater omentum on the GC side (Fig. 1a,b). The branch of the right gastroepiploic artery is longer at the PW than at the AW^9^.

**Figure 3:**
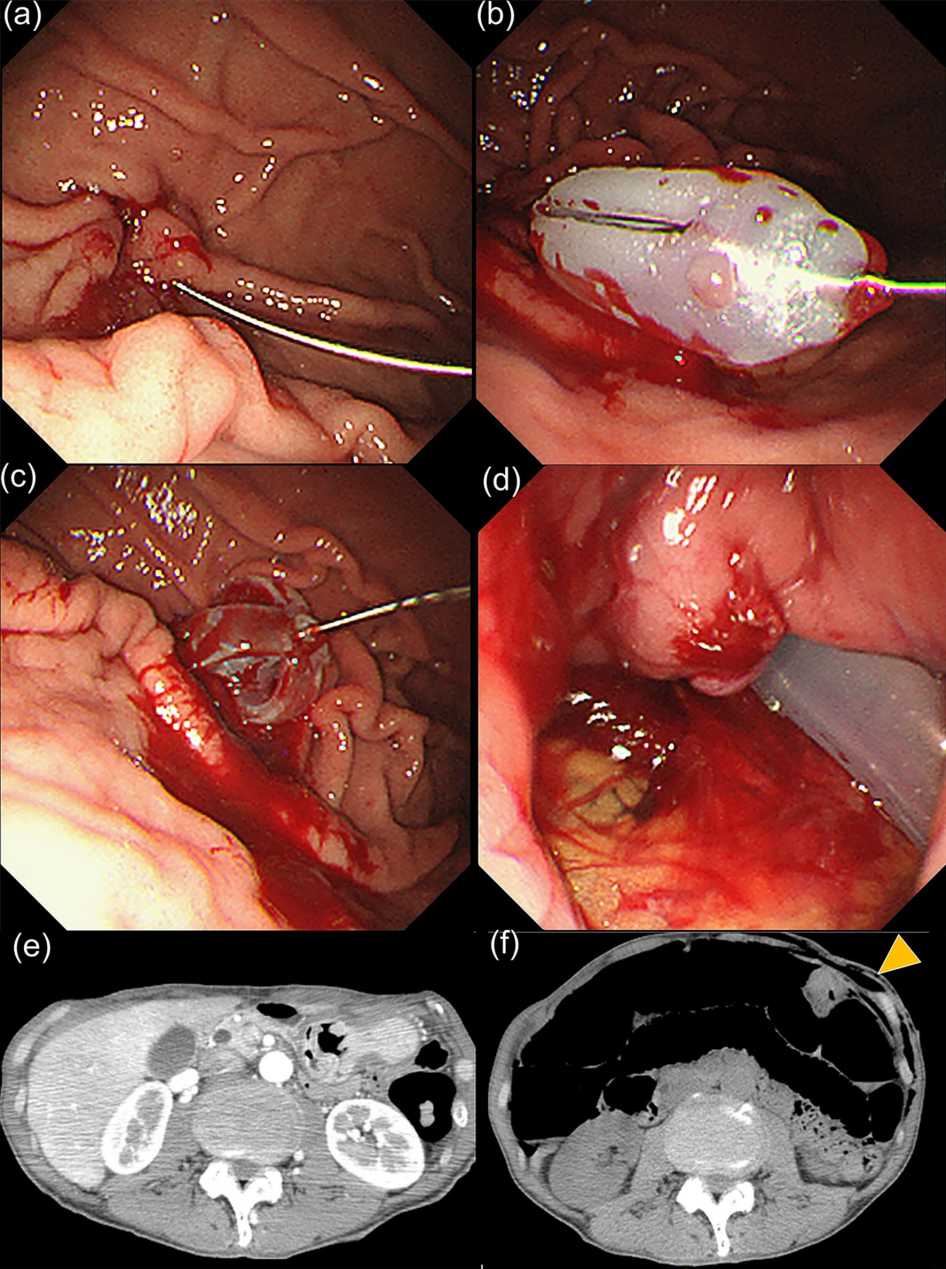
Endoscopic and CT images before and after PEG construction in case 2. (**a**) Three-point gastric wall fixation was performed near the PW of the GC. (**b**) As it was difficult to insert the dilator, a skin incision was added for placement of the gastrostomy tube into the stomach. (**c**) Bleeding occurred immediately after the procedure. (**d**) Gastric perforation was detected. (**e**) Before PEG construction, there was no intestinal interference between the stomach and abdominal wall. (**f**) After discontinuing PEG construction, there was free air in the abdominal cavity and subcutaneous emphysema in the abdominal wall.

Case 2 was a 60-year-old male patient. PEG was indicated for nutritional management because of food intake difficulty due to pharyngeal cancer growth. Blood tests were unremarkable except for an albumin level of 3.5 g/dL. Contrast-enhanced CT detected no interfering organs between the stomach and abdominal wall (Fig. 3e). The gastric AW was distant from the abdominal wall (Fig. 1f), which necessitated PEG insertion at the PW of the GC. The usual four-point gastric wall fixation was abandoned for three-point fixation (Figs. 1f, 3a). As the insertion dilator was difficult to place after the first skin incision, an additional incision was made prior to gastrostomy tube insertion into the stomach using a guide wire (Fig. 3a,b). Acute bleeding occurred soon after (Fig. 3c). Upon noticing a gastric perforation after washing away the blood (Fig. 3d), we immediately removed the gastrostomy tube and terminated the endoscopic procedure. After discontinuation, CT revealed free air in the abdominal cavity and subcutaneous emphysema in the abdominal wall (Fig. 3f) requiring urgent surgery. A hematoma was detected around the stomach wall, and the two-centimeter incision in the short-axis direction to the PW of the GC was visualized. We usually puncture the abdominal wall with a dilator or PEG tube in the AW of the stomach (Fig. 1b). If the stomach is rotated in the long axis, it is difficult to insert the dilator vertically into the AW of the middle body of the stomach (Fig. 1b). In this case, the dilator was likely inserted towards the PW site, and the stomach wall firstly became torn in the short-axis direction (Fig. 1f). Afterwards, the PEG tube was inserted nearby to the damaged stomach wall in the PW (Fig. 1g). Finally, the damaged stomach wall became torn due to the traction compression for bleeding (Fig. 1g).

## Discussion

This study clarified that PEG tube insertion at the PW of the GC was an independent risk factor for early acute complications of the PEG procedure and described two cases of early acute complications recently encountered at our institution.

The overall complication rates at 2 weeks and 3 months after PEG construction have been reported as 39% and 27%, respectively^10^. Jafari et al. observed that 3.9% of 641 PEG cases displayed serious complications, including perforation, intra-abdominal abscess, and buried bumper syndrome^11^. Our cohort contained 80 complication cases in total (14.8%), 62 of which (11.5%) occurring within 30 days after the PEG procedure. Those included early acute complications in 42 cases (7.8%) that consisted mainly of wound bleeding (32 cases; 5.9%) and arterial bleeding (five cases; 0.9%). Sin et al. reported bleeding as the most frequent acute complication (12.8%) using pull-type (11.8%) or introducer-type (14.3%) gastrostomy^12^. We routinely used introducer-type gastrostomy to conduct PEG in this study. The above data suggest that clinicians should bear complications in mind, specifically bleeding, when performing the PEG procedure.

The mild bleeding sometimes encountered near the PEG wound site is typically managed by conservative treatment, such as simple pressure to the wound. Severe hemorrhage is rare, but can occur by vascular puncture or damage^13–15^ as seen in case 1. Indeed, we observed arterial bleeding in 0.9% (5/540) of cases after PEG tube insertion, two of which needed surgical treatment. Regarding bleeding complications, specifically with blood vessel injury as the suspected cause, a list of previous reports have been summarized in Table 4. The arteries surrounding the stomach involved in such injuries included the gastroepiploic artery^16,17^, gastric artery^15,18–20^, and splenic and superior mesenteric artery^14,21, 22^. The artery injuries were caused by stomach rotation^17^, over-inflation of the stomach resulting in rotation^15^, multiple punctures^18,19,22^, loss of traction and torsional stress between ligaments and vessels^20^, and fibrosis and adhesions around the stomach due to postoperative cholecystectomy^14,21^. Our study revealed that PEG procedures at the PW of the GC were significantly associated with acute complications, including arterial injury. This might have been on account that major arteries run near this area to elevate the risk of blood vessel injury. Clinicians should pay careful attention to arterial complications after PEG at the PW of the GC.

**Table 4:** Reported cases of vessel injury associated with PEG insertion.

Injured blood vessel	PEG location site	Diagnosis time after PEG procedure	Number of insertions	Cause	Treatment	Outcome	References
GEP	–	–	–	–	OP	–	^16^
Rt and Lt GEP	GC	6.5 days	–	Rotation of the stomach	EM	Recovered	^17^
GA	GC	0 days	–	Rotation of the stomach due to over-inflation	OP	–	^15^
LGA	AW	50 min	3	(1) Three-point gastric fixation (2) Multiple insertions	EM	Died	^18^
LGA	AW	3 days	4	Multiple insertions	EM	Recovered	^19^
SGV	–	12 h	–	Traction and torsional stress on the spleen along the gastro-splenic ligament and splenic vessels derived from maximal gastric insufflation	OP	Recovered	^20^
SV and SMV	AW	2.5 h	2	(1) Long length of needle (7 cm) (2) Vertical or oblique displacement of needle (3) Fibrosis and adhesions between liver and stomach due to postoperative cholecystectomy	OP	Died	^14^
B-SA	11 cm proximal to the pylorus	Several hours	2	Fibrosis and adhesions between liver and stomach due to postoperative cholecystectomy	CS	Died	^21^
PB-SMA	LC of AW	1 day	2	(1) Multiple insertions (2) Deep insertion	EM	Recovered	^22^

*AW* anterior wall, *B-SA* branch of splenic artery, *CS* conservative treatment, *EM* embolization, *GA* gastric artery, *GC* greater curvature, *GEP* gastroepiploic artery, *LC* lesser curvature, *LGA* left gastric artery, *OP* operation, *PB-SMA* pancreatic branch of the superior mesenteric artery, *PEG* percutaneous endoscopic gastrostomy, *Rt and Lt GEP* right and left gastroepiploic artery, *SGV* short gastric vessel, *SV and SMV* splenic and superior mesenteric vein.

– Not described.

Previous cases of gastric perforation have been linked to insufficient gastric wall fixation^18,23^. In case 2, the operator had initially attempted to make four-point gastropexy as a square (Supplementary Fig. S1b). However, during the fourth point puncture, the operator noticed that the needle was tangentially inserted into the gastric wall, and not into the stomach. The operator then proceeded to puncture the dilator with under incomplete fixation in the form of an isosceles triangle instead of an equilateral one (Supplementary Fig. S1a,c). At that point, alternative methods should have been considered for a safer approach. The puncture power was tangentially directed along the spherical surface of the stomach wall of the PW of GC and was not transmitted perpendicularly to the stomach wall, creating a risk of stomach wall injury. After fixation, the thread of the sutures should be pulled in the opposite direction of the dilation/puncture to give counter-traction for greater safety, which was done in the presented cases. To avoid the unnecessary puncture of gastroepiploic blood vessels, selecting three gastropexy points in the pattern of an equilateral triangle instead of four points may also reduce complications during PEG tube insertion at the PW of the GC, especially for cases of esophageal hernia. In addition, direct percutaneous endoscopic jejunostomy^24^, percutaneous transesophageal gastro-tubing^25^, safe gastric puncture point by a plain abdominal film with air insufflation technique^26^, CT-guided PEG^27^, and laparoscopy-assisted introducer PEG^28,29^ should be considered as alternative methods to achieve long-term enteral nutrition. It is important for clinicians to select from push and pull methods as flexibly as possible according to the patient’s condition in order to reduce the risk of severe complications.

Lastly, the use of aspirin or clopidogrel is not reportedly associated with an increased risk of bleeding^30–32^. However, no studies have examined the risk of PEG construction in patients on prasugrel, ticagrelor, or direct oral anticoagulants^13^. Since PEG candidates are also indicated for those medicines in the clinical setting, the frequency and dose of such drugs should be considered to reduce possible bleeding complications.

This study had several limitations. First, as the number of patients receiving PEG at the PW of the GC was small for multivariate analysis, additional cases are needed to statistically validate our results. Second, the final clinical outcome of some complications was not recorded in this retrospective study due to patient transfer to another hospital. However, we received no reports of severe PEG-associated complications from the subsequent institutions. Larger prospective investigations are required to clarify the outcomes of PEG procedures.

In conclusion, this 12-year analysis on PEG insertion site identified PEG at the PW of the GC as an independent risk factor for acute complications and described the details of two cases of severe complications involving this site that required urgent additional treatment. If unavoidable, PEG at the PW of the GC should be accompanied with careful observation for early acute bleeding complications.

## Material and methods

### Patients and study design

A retrospective review was performed using the medical records of patients who underwent PEG at Shinshu University Hospital (Nagano, Japan) and Matsumoto City Hospital (Nagano, Japan) during an approximately 12-year period between April 2008 and December 2019. A total of 570 patients were initially targeted for comparisons of PEG procedures based on medical charts, endoscopic reports, and endoscopic and radiological images. Thirty cases were excluded for the following reasons: negative finger sign for safe puncture (13 cases), general condition exacerbation (11 cases), lack of endoscopic findings (two cases), superimposition with gastric cancer on the puncture route (one case), gastric ulcer scar on the puncture route (one case), non-passage of the esophagogastroduodenoscopy scope through the esophagus (one case), and no consent before procedure (one case). Ultimately, 540 patients were included in the analysis. Informed consent was obtained from all subjects or their legal guardians when appropriate. This study was conducted in accordance with the principles of the 1975 Declaration of Helsinki and approved by the institutional review board of Shinshu University School of Medicine (approval number: 4048) and Matsumoto City Hospital (approval number: 022).

### Definitions of complications

Complications were divided into two groups based on onset time being within 30 days or 30 days or more after the PEG procedure (Table 2). Early acute complications were defined as bleeding or perforation related to PEG and included early severe complications according to a previous report^5^. Severe acute complications were defined as those requiring surgical intervention within 30 days after PEG construction.

### Determination of PEG tube insertion site

We defined the PEG tube insertion site from endoscopic images based on a previous study^8^.

### PEG procedure

All PEG procedures were performed by an endoscopist, an assistant doctor who directly participated in the surgery, and 1–2 nurses. An introducer-type gastrostomy set (PEG-24-introducer-type [Ideal button]; Olympus, Tokyo, Japan) and an upper gastrointestinal endoscope (GIF-Q260, GIF-XP260NS, or GIF-XP260; Olympus, Tokyo, Japan) were used following the administration of lidocaine spray as an oral local anesthesia and midazolam and/or pentazocine for sedation. Before PEG tube insertion, a finger sign by pressing with a finger on the body surface was conducted and endoscopically confirmed by compression in the gastric lumen^33^. Transillumination was also performed, with the light identified through the abdominal wall to determine the best site for PEG tube insertion^33^. The skin was disinfected by povidone-iodine and lidocaine was infiltrated into the skin with a 23-gauge needle. Four-point gastric wall fixation was performed before placement of the PEG tube, which was selected to be as long as possible. Then, a skin incision was made in the center of the four-point gastric fixation, a hole was made with a puncturing needle, and a guide wire was detained under endoscopic vision into the stomach. A dilator was inserted along the guide wire, the length of the gastrostomy was measured, and the gastrostomy tube was inserted after removing the dilator. The procedure was completed after confirming hemostasis. Prophylactic antibiotics (cefazolin sodium 1 g/dose twice a day on day 1 and once on the following day) were routinely administered at the time of each procedure. The PEG insertion site was disinfected with povidone-iodine for 1 week after the procedure.

### Statistical analysis

Statistical analysis was performed by StatFlex software version 7.0.10 (Artec, Osaka, Japan). Continuous variables are presented as the median and lower to upper limit, and categorical variables are expressed as the frequency (%). The Mann–Whitney U-test was used to analyze continuous variables. The chi-square test was employed for categorical variable comparisons, with Fisher’s exact probability test adopted for groups with fewer than five samples. Statistically significant variables in the univariate model were subsequently used in multivariate analysis to identify independent predictors of complications. The odds ratio (OR) and 95% confidence interval (CI) were obtained by means of univariate and multivariate models. A *p*-value of < 0.05 was considered to indicate a statistically significant difference.

## Supplementary information

Supplementary Information.
