# Supplementary material for: Relationship of early acute complications and insertion site in push method percutaneous endoscopic gastrostomy
Source: Sci Rep. 2020 Nov 25;10:20551. doi: 10.1038/s41598-020-77553-6 (PMC7689522; doi:10.1038/s41598-020-77553-6)

Relationship of early acute complications and insertion site in push method percutaneous endoscopic gastrostomy

Hiroshi Suzuki^1$^, Satoru Joshita^1$^, Tadanobu Nagaya^1＊^, Koichi Sato^1^, Akihiro Ito^2^,　Tomoaki Suga^1^, Takeji Umemura^1,3^

1.　Department of Medicine, Division of Gastroenterology, Shinshu University School of Medicine, Matsumoto, Japan

2.　Department of Gastroenterology, Matsumoto City Hospital, Matsumoto, Japan

3.　Department of Life Innovation, Institute for Biomedical Sciences, Shinshu University, Matsumoto, Japan

Table of contents

Supplementary Table 1…………………………………………………………… 2

Supplementary Figure 1………………………………………………………….. 3

**Supplementary Table 1: Independent factors associated with early acute complications in two additional models**

| Model 2 |  |  | Model 3 |  |  |
| --- | --- | --- | --- | --- | --- |
| Included factors | *p*-value | OR (95%CI) | Included factors | *p*-value | OR (95%CI) |
| Anti-platelet or  anti-coagulant agents | 0.039 | 2.06 (1.04-4.07) | Hemoglobin | 0.041 | 0.85 (0.73-0.99) |
| PW of GC | 0.0004 | 9.01 (2.68-30.26) | PW of GC | 0.0002 | 9.99 (2.96-33.70) |

Abbreviations: CI, confidence interval; GC, greater curvature; OR, odds ratio; PW, posterior wall.

Supplementary Figure 1.

(a)　Schematic of equilateral triangle three-point fixation. (b) Schematic of square four-point fixation. (c) Schematic of isosceles triangle three-point gastric wall fixation in case 2. Puncture power was tangentially directed along the spherical surface of the stomach wall of the PW of the GC and was not transmitted perpendicularly to the stomach wall, creating the risk of stomach wall injury.


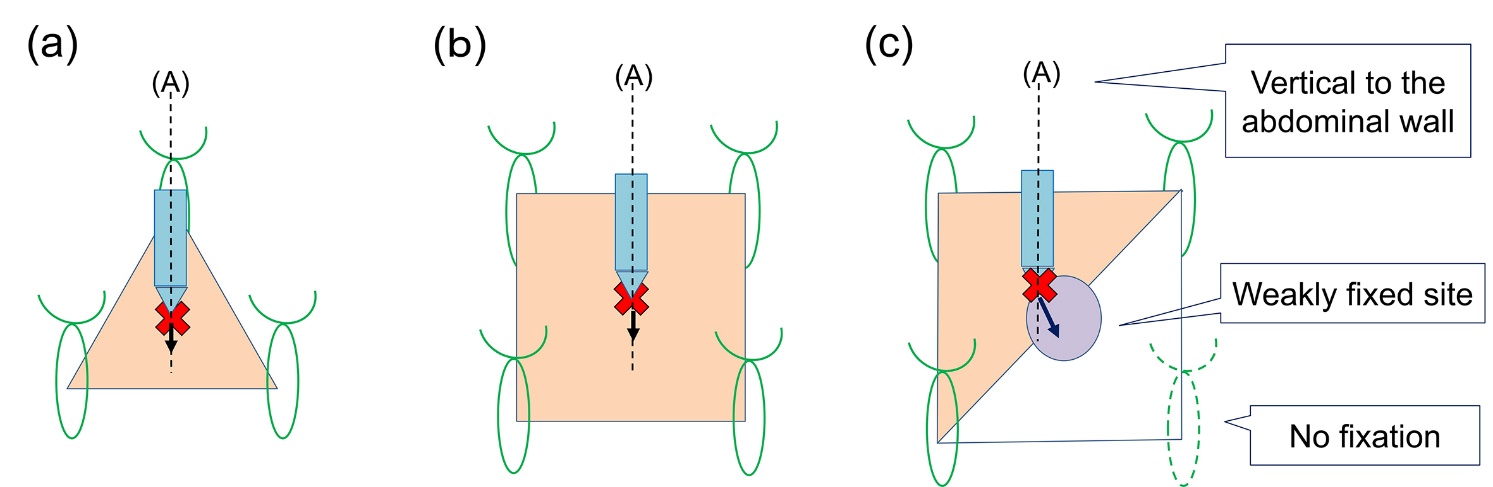

Supplement: Supplementary file 1 — Supplementary Information. [file 41598_2020_77553_MOESM1_ESM.docx]
